# Salvage prostate intensity modulated radiation therapy after cryotherapy failure

**DOI:** 10.1038/s41598-024-59406-8

**Published:** 2024-04-21

**Authors:** Tanguy Perennec, Maximilien Rogé, Jean-François Hetet, Philippe Colls, Valentine Guimas, Emmanuel Rio, Loïg Vaugier, Stéphane Supiot

**Affiliations:** 1 Department of Radiation Oncology, Institut de Cancérologie de L’Ouest, Bd Professeur Jacques Monod, 44800 Saint-Herblain, France; 2 Department of Radiation Oncology, Centre Henri Becquerel, 1 Rue d’Amiens, 76000 Rouen, France; 3 Department of Urology, Clinique Jules Verne, 2-4 Route de Paris, 44300 Nantes, France

**Keywords:** Prostate cancer, Salvage radiotherapy, Cryoablation, Biochemical relapse, Toxicity, Prostate cancer, Radiotherapy

## Abstract

Cryotherapy is an ablative therapy that can be used to treat localized prostate cancer. In case of recurrence, treatment options are not well-defined, and their outcomes are unknown. We therefore collected all patients treated with radiotherapy after cryotherapy for prostate cancer recurrence in Nantes (France) between 2012 and 2019. We identified ten patients. After a median follow-up of 5 years, two patients presented late grade 3 toxicities; one patient presented a grade 3 rectal hemorrhage, and one had a grade 3 hematuria. Two patients relapsed at 61 and 62 months, and three patients died of other causes. Radiotherapy to treat local prostate cancer recurrence after cryotherapy seems feasible and effective in local control. These results do not allow us to recommend this technique in current practice but are encouraging for the conduct of prospective trials.

## Introduction

Cryotherapy is a minimally invasive procedure that aims to destroy tumor cells by exposing them to extremely low temperatures (− 40 to − 50 °C). Cryotherapy induces cell death through several biological mechanisms. First, through the formation of ice crystals in the extracellular spaces, which creates a hyperosmotic extracellular environment, and then through cold injuries (endothelial damage with increased capillary wall permeability, edema, platelet aggregation, and microthrombus formation) causing microcirculatory failure. Finally, mainly in the peripheral region of the cryotherapy zone, cell apoptosis contributes to killing the surviving cells. This latter mechanism primarily occurs after the tissue is reheated^1,2^.

Third-generation cryotherapy brought technical advances, such as the use of thermosensors and the application of gas-based cryosurgery, which improve its efficacy and significantly reduce complications such as incontinence, urethral sloughing, and rectourethral fistulas compared to previous generations^3^. European guidelines now recognize this technique as an early-stage prostate cancer treatment option, but only within clinical trials^4^. Cryotherapy could also be a salvage treatment option for cancer recurrence after radiotherapy^5^. However, the National Comprehensive Cancer Network (NCCN) guidelines do not currently endorse cryotherapy as a valid treatment option^6^.

Biochemical relapse rates after primary cryotherapy are reported to range from 55 to 90% at five years^7–9^. Those relapses may be local in many patients treated by cryotherapy. For example, Jones et al. reported a 38% rate of positive post-treatment biopsies in patients who experienced biochemical relapse after cryotherapy^10^. The treatment of local prostate cancer recurrence after cryotherapy is challenging since the optimal management is unknown. Treatment options include androgen deprivation therapy (ADT) palliative therapy, a new round of cryotherapy, radical prostatectomy, and radiotherapy. Current literature on salvage radiotherapy (SRT) after cryotherapy failure is limited to small, single-center retrospective studies. In these studies, approximately half of the patients were treated with outdated radiotherapy techniques, such as 2D or 3D-conformal^9,11^. Even among patients treated with modern radiotherapy techniques, such as Intensity-modulated Radiation Therapy (IMRT) or Stereotactic Body Radiotherapy (SBRT), there is considerable variability in SRT modalities^12–15^**.**

Therefore, the aim of our study is to report the oncological outcomes and toxicities of a series of patients treated with IMRT following cryotherapy.

## Materials and methods

### Study design and participants

This single-center retrospective study was conducted according to the guidelines of the Declaration of Helsinki. It was approved by the Institutional Ethics Committee of Angers Hospital in France (protocol code 2022/114, date of approval 08 July 2022). We used the institutional registry database to identify patients meeting the inclusion criteria: men with a histologically confirmed local prostate cancer history, treated initially by cryotherapy, who then underwent salvage radiotherapy after biochemical relapse between January 2014 and December 2021. Patients were excluded if they had metastatic lesions diagnosed before completing radiotherapy or if they underwent surgical treatment for prostate cancer.

### Cryotherapy

All patients received standard third-generation cryotherapy (including high-precision real-time transrectal ultrasound, use of urethral warming, multiple thermocouples through the prostate, and multiple cryoprobes) as their primary treatment for localized prostate cancer. The procedure was not associated with transurthral resection. Biological relapse was subsequently diagnosed with PSA rise on two biological samples. The isolated local recurrence was confirmed with prostate biopsy in all cases. Pathological grading of these biopsies was always performed. In the case of partial cryotherapy, the results of the biopsies were always based on the tumoral characteristics on the same side as prior cryotherapy. Furthermore, the distant metastatic staging was always performed and was negative for metastatic disease.

### Radiotherapy

All patients underwent computed tomographic (CT) simulation with 3 mm thick slices. For certain patients, a prostate MRI was also conducted for enhanced delineation. Prostate Clinical Target Volume (CTV) corresponded to the whole prostate and the seminal vesicles, in cases where they were treated. A 3D margin of 10 mm (except in posterior: 5 to 7 mm) was added to the Prostate CTV to form the Planning Target Volume (Prostate PTV). In some patients, pelvic lymph nodes were contoured, and a margin of 5 mm was added to define the PTV.

All patients were treated using Intensity Modulated Radiation Therapy (IMRT) on Helical Tomotherapy or linear accelerator (LINAC)-based IMRT (Truebeam) with a non-empty bladder and an empty rectum. Daily verification of positioning using IGRT (image-guided radiation therapy) was performed.

### Follow-up

Toxicities were reported using the Common Terminology Criteria for Adverse Effects (CTCAE) version 5.0^16^. We defined residual toxicity after cryotherapy as any toxicity that persists beyond one month after the cryotherapy procedure. Toxicities were defined as *acute* if occurring in the first six months after completion of SRT, *late* if afterwards.

We used the definition proposed by RTOG-ASTRO (Radiation Therapy Oncology Group- American Society for Therapeutic Radiology and Oncology) Phoenix consensus to define the biochemical recurrence (PSA Nadir + 2 ng/mL^17^.

Patients were followed using PSA measurement and clinical examination commonly every six months. In case of biochemical recurrence, patients underwent Choline or PSMA PET-CT.

### Statistical analysis

We presented all characteristics for each patient and describe the population with median and range for continuous data and number and percentage for categorical data. Biochemical Disease-Free Survival (bDFS) was defined by the occurrence of biochemical recurrence or the occurrence of death from any causes. The curve has been obtained with Kaplan–Meier method. Median time of follow up has been estimated using reverse Kaplan–Meier method.

### Informed consent.

Informed consent was obtained from all individual participants included in the study.

### Institutional review board statement

The study was conducted according to the guidelines of the Declaration of Helsinki and was approved by the Institutional Ethics Committee of Angers Hospital in France (protocol code 2022/114, date of approval 8th of July 2022).

## Results

### Patient characteristics before radiotherapy

We identified ten patients who met the inclusion criteria in the institutional registry database. Patients were all diagnosed with prostate cancer with 12 cores biopsies. According to the D’Amico classification, three patients (30%) were low risk, six (60%) were intermediate risk, and one (10%) was high risk.

Cryotherapy the initial treatment for prostate cancer in all patients, and only one cycle was performed. The justification for such treatment was reported in height patients. Six patients were having a poor general condition. Cryotherapy was therefore proposed as an alternative to conventional treatments. Two patients (patients 1 and 3) were described as having a very low risk and cryotherapy was proposed as an alternative to active surveillance.

The prostate volume treated during cryotherapy was the entire gland in five patients, while only one lobe was targeted in the other half. Three patients experienced residual genitourinary (GU) toxicities after cryotherapy, (one grade 1 and two grade 2). No patients received ADT during the cryotherapy or the year following the cryotherapy. Prostate cancer recurrence after cryotherapy failure was treated with salvage radiotherapy (SRT) between April 2012 and April 2019. The median age of patients was 76 (63—83) years. The median PSA level was 6.4 ng/mL (2.1—11.2). The median time from cryotherapy to SRT was 34 months (range 13–92 months). Extended patient and prostate cancer characteristics can be found in Table 1.

**Table 1:** Patients’ characteristics at prostate cancer diagnosis, cryotherapy and before salvage radiotherapy.

No	Diagnosis				Cryotherapy			SRT				
	Age (years)	Initial Gleason	PSA (ng/mL)	cT stage	Cryotherapy technique	Number of needles used	Residual toxicity	Age (years)	Gleason	PSA (ng/mL)	PSAdt (month)	Time Cryo-SRT (month)
1	74	6 (3 + 3)	7.6	T1c	Whole	8	0	79	6 (3 + 3)	4.6	114	48
2	76	7 (3 + 4)	15.0	T2a	Whole	6	Urinary tract obstruction G1	80	7 (4 + 3)	10.1	10,6	34
3	72	6 (3 + 3)	7.4	T1c	Whole	9	0	75	7 (3 + 4)	3.4	3,1	35
4	69	7 (3 + 4)	8.1	T1c	Whole	7	Urinary tract obstruction G2	74	7 (3 + 4)	7.9	13	57
5	62	6 (3 + 3)	7.5	T2a	Whole	8	Urinary frequency G2	63	7 (4 + 3)	4.9	5,9	13
6	74	7 (3 + 4)	7.9	T2a	Partial	5	0	77	7 (4 + 3)	2.1*	NA*	31
7	75	7 (3 + 4)	9.4	T2c	Partial	4	0	78	8 (4 + 4)	5.2	8,3	33
8	73	7 (3 + 4)	7.8	T1c	Partial	5	0	75	7 (3 + 4)	10.8	13,8	20
9	71	8 (4 + 4)	9.7	T2a	Partial	3	0	75	7 (4 + 3)	11.2	10,3	27
10	74	7 (3 + 4)	8.7	T2a	Partial	3	0	83	7 (4 + 3)	9.9	44,5	92

SRT, Salvage Radiotherapy; PSAdt, PSA doubling time; G1, Grade 1; G2, Grade 2.

*This patient received bicalutamide before performing his pre-SRT PSA, his PSA-doubling time is non-computable.

Radiotherapy characteristics and toxicities are presented in Table 2. All patients received a normofractionated treatment with a median dose to the prostate of 76 Gy (range 74–78 Gy) in 34 to 39 fractions of 2.0 Gy to 2.2 Gy. Four patients (40%) also received pelvic lymph node radiotherapy.

**Table 2:** Details on salvage therapy.

	CTV	Dose (Gy)	Number of fractions	Dose Pelvic LN (Gy)	ADT before SRT (day)	ADT during SRT	Duration of ADT (month)	GU pre-RT	GI toxicity	GU toxicity
1	Prostate + SV	77	37	55	0	Yes	32	0	0	0
2	Prostate alone	78	39	0	0	No	0	Urinary tract obstruction G1	0	Urinary tract obstruction G2, Urinary urgency G1, Urinary frequency G2
3	Prostate alone	76	38	0	0	No	0	0	0	0
4	Prostate alone	76	38	0	0	No	0	Urinary frequency G2	Rectal hemorrhages G1	Urinary frequency G1
5	Prostate alone	76	38	0	47	Yes	4	Urinary tract obstruction G2	0	Incontinence G2, Urinary frequency G1, Hematuria G3
6	Prostate + SV	74,8	34	54,8	38	Yes	41	0	Rectal hemorrhage G3	Urinary frequency G1
7	Prostate alone	76	38	0	75	Yes	36	0	0	Urinary frequency G1
8	Prostate + SV	74,8	34	54,4	36	Yes	12	0	0	Urinary frequency G1
9	Prostate + SV	74	37	55,5	47	Yes	23	0	0	0
10	Prostate + SV	74	37	0	43	Yes	24	0	0	Urinary frequency G1

CTV, clinical target volume; SV, Seminal vesicles; Gy, Grays; ADT, androgen deprivation therapy; LN, lymph nodes; SRT, salvage radiation therapy; GU, genito-urinary; GI, gastro-intestinal; G1/2/3, grade 1/2/3.

Two patients presented late grade 3 toxicities, a grade 3 rectal hemorrhage (requiring transfusion) and a grade 3 hematuria (macroscopic and needing a transfusion). One should notice that the patient who experienced grade 3 hematuria was undergoing treatment with vitamin K antagonist for atrial fibrillation. He had persistent and fluctuating macroscopic haematuria during the entire follow-up period. Initially intense and daily, with clots, they became minimal and transient (with hematuria-free periods lasting several months) from the year following radiotherapy. Grade 3 rectal toxicity consisted of one episode of rectal discharge requiring transfusion, with no recurrence.

Apart from these two toxicities, we noted only one other late digestive toxicity (grade 1). The other urinary toxicities were only graded 1 except for one patient who presented with urinary tract obstruction and urinary urgency grade 2. No patient had residual GI toxicity from cryotherapy and three patients already had some urinary toxicity with radiotherapy.

**Figure 1:**
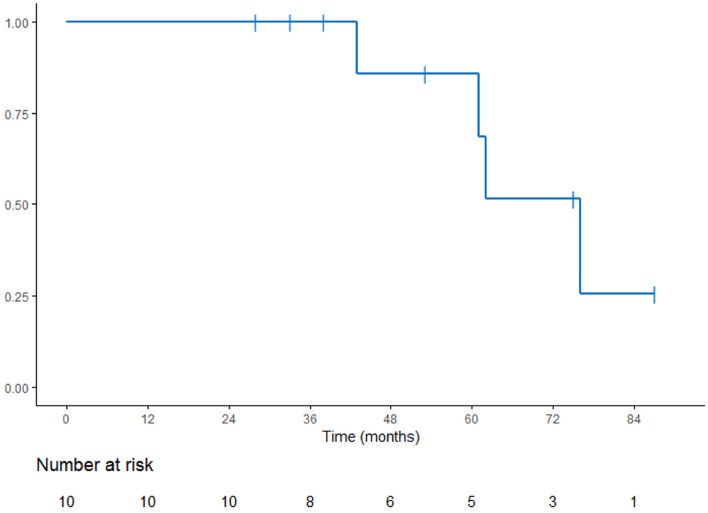
Biochemical Disease-Free Survival (bDFS).

**Figure 2:**
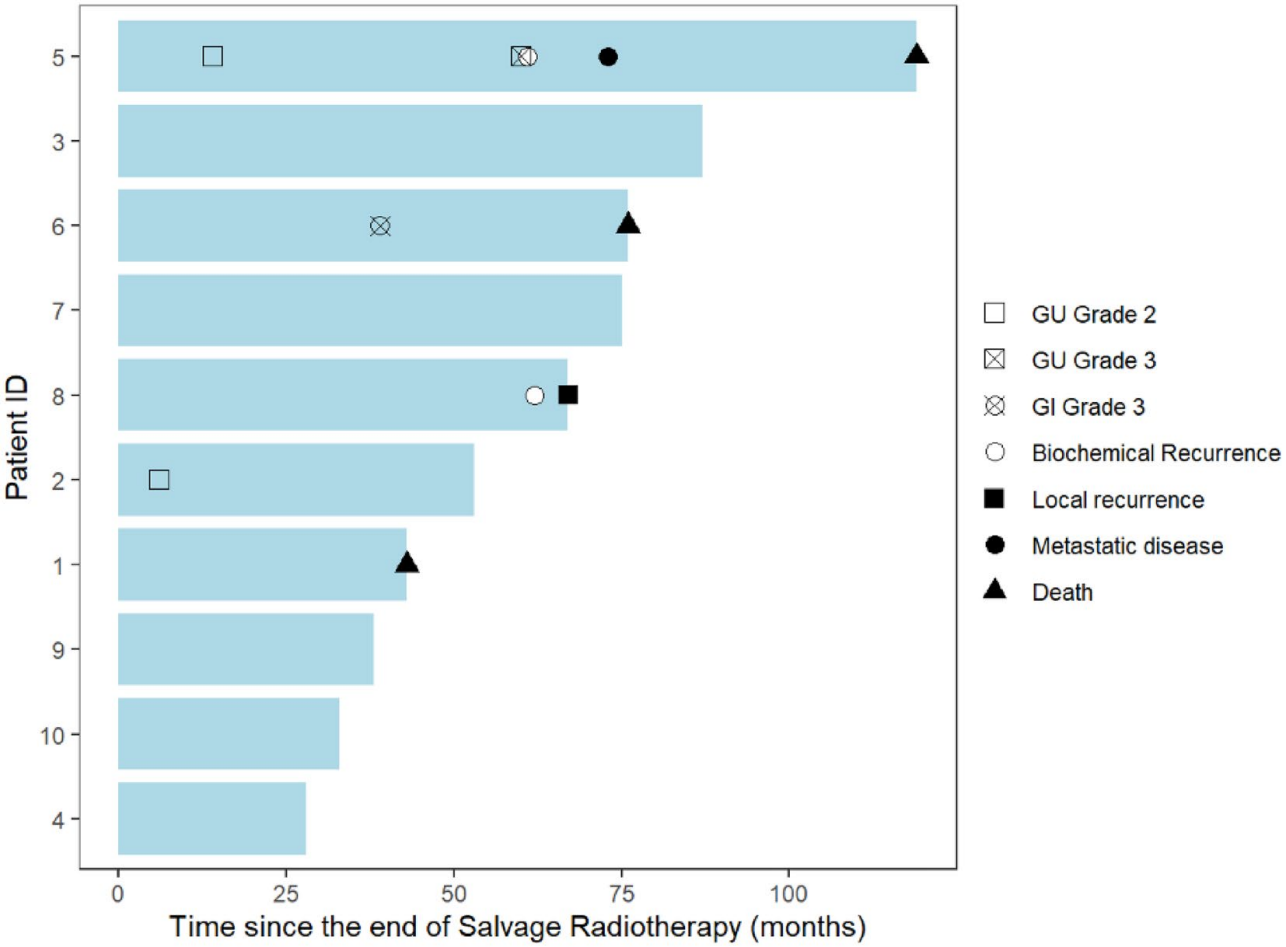
Swimmer Plot. GU: Genito-urinary; GI: Gastro-intestinal.

After a median follow-up of 60 months (28—119) after SRT, two patients experienced biochemical recurrence at respectively 61 and 62 months. Median biochemical relapse-free survival time was 76 months (Fig. 1). One had a local recurrence diagnosed with PET choline at 67 months and the other one experienced a distant progression diagnosed with PET PSMA at 73 months and died 119 months after the end of SRT. Two patients, without any prostate cancer recurrence, died from other causes (esophagus cancer and non-cancerous disease). Figure 2 summarizes each outcome and toxicities.

## Discussion

Our study shows that salvage IMRT for a local recurrence of prostate cancer previously treated with cryotherapy seems feasible but could generate some grade 3 toxicities. On the other hand, it might be a good option in terms of cancer control. Indeed, none of the ten patients described in our paper experienced a disease progression within the first two years.

In our series, the treatments performed were highly heterogeneous (in terms of volumes treated, doses delivered, addition of hormone therapy, etc.), as were the clinical situations of each patient (medical history, cancer characteristics, urinary and digestive functions). This is an undeniable limitation to the interpretation of results and does not allow conclusions to be drawn on possible interaction factors with toxicity or recurrence-free survival. However, even if the presence of severe toxicity cannot be directly attributed to a particular strategy, it prompts the utmost caution when considering post-cryotherapy treatment with radiotherapy.

Specifically, although we cannot conclude on the relationship between the dose prescribed or the volumes treated (in particular lymph node irradiation), it seems legitimate to question the relevance of lymph node irradiation. In our series, four patients underwent lymph node irradiation. As this series was retrospective, the indications for lymph node irradiation were at the discretion of each physician, and are, once again, not homogeneous. Given the significant toxicity experienced by some patients, it is advisable, when considering post-cryotherapy irradiation, to ensure that lymph node irradiation is relevant and that it does not significantly alter dosimetry at the rectal level. Also, salvage radiotherapy following cryotherapy is a practice that we consider to be risky, and it should be carried out under the best possible conditions, particularly in terms of imaging, where MRI and PET should be performed. These examinations were not systematically carried out in our series.

Alternatives to radiotherapy are the initiation of palliative ADT and other salvage local. ADT has adverse effects affecting the quality of life (such as hot flushes, reduced libido, and increased fracture risk)^18^. On the other hand, side effects and effectiveness of salvage treatments are not well known. Salvage radiotherapy represents a new opportunity for curative treatment before considering approaches such as hormone therapy.

The lack of a standard endpoint and the lack of individual data in the literature makes it difficult to put our results into perspective. Nevertheless, It seems that our study is consistent with previous published series^19^. Lischalk et al. recently published an extensive series of patients (n = 51) who underwent stereotactic body radiotherapy after cryotherapy failure and provided detailed oncological outcomes^12^. In this study, authors reported a median biochemical relapse-free survival of 66 months vs. 76 months in our study. Thus, it should be noted that the patients included in their study had higher mean PSA level before SRT than in our study (11.3 ng/mL versus 7.0 ng/mL). In the Lischalk study, a minority of patients (35%) received ADT, compared to 70% in our study. Therefore, caution should be exercised in interpreting differences in biochemical relapse rates.

In another study published by Hopper et al.^14^ in 2017, a 5-year biochemical disease-free survival of 75% was reported for eight patients, which is better than the rate reported in our study. However, due to the small number of patients and the limited follow-up time, no firm conclusions can be drawn. The population of study was similar to ours.

In our study, we reported 2 grade 3 toxicities (one rectal hemorrhage and one hematuria). Those severe complications had not been reported in previous studies. Choi et al. published in 2013 a 7-patient series treated with salvage IMRT after cryotherapy and did not report any grade 3 complications^15^ as well as the Hopper study (with 8 patients)^14^***.*** In Lischalk study, only one patient (2%) experienced a grade 3 GU toxicity (urinary tract obstruction), 51 months after SRT. It should be noticed that grade 3 toxicities may occur quite late after radiotherapy completion. Here, we observed grade 3 toxicity at 39 and 61 months respectively, raising the concern that the median follow-up in the Choi (31 months), Jiang (23 months) and Lischalk (40 months) studies might have been insufficient to detect these complications and may have underestimated their incidence. Cryotherapy is sometimes offered to elderly, or patients with comorbidities, which is known to be associated to more complication rate with radiation therapy^20^.

There is no consensus on the best treatment for prostate cancer recurrence after cryotherapy, and we have not identified any prospective or randomized trials being enrolled or analyzed that evaluate and compare different strategies. Thus, our study provides new data, with the longest available follow-up in the literature, enhancing the evidence for the efficacy of salvage radiotherapy. On the other hand, these results call for great caution, given the serious long-term toxicity observed.

## Data Availability

All data generated and analyzed during this study are included in this published.
